# P-174. Exploring Healthcare Seeking Patterns for Nipah-like Illnesses in Bangladesh: Findings from a Community-based Study

**DOI:** 10.1093/ofid/ofaf695.398

**Published:** 2026-01-11

**Authors:** Dewan Imtiaz Rahman, Wasik Rahman Aquib, Farzana Fariha, Tonmoy Sarkar, Fatema Akther Ema, Utpal K Mondal, Mohammad Rezaul Karim, Shadman Sakib Choudhury, Kamal Ibne Amin Chowdhury, Anika Farzin, Faruq Abdulla, Muhammad Rashedul Alam, Kajal Chandra Banik, Shah Jawad Bin Mahmood, Arifur Rahman Bablu, Arifa Nazneen, Ayesha Siddika, Mohammad Enayet Hossain, Ahmed Nawsher Alam, Sharmin Sultana, Trevor Shoemaker, Christina Spiropoulou, Mohammed Ziaur Rahman, Sayera Banu, Tahmina Shirin, Joel M Montgomery, Syed Moinuddin Satter

**Affiliations:** icddr,b, Dhaka, Dhaka, Bangladesh; icddr,b International Centre for Diarrhoeal Disease Research, Bangladesh, Dhaka, Dhaka, Bangladesh; icddr,b, Dhaka, Dhaka, Bangladesh; icddr,b International Centre for Diarrhoeal Disease Research, Bangladesh, Dhaka, Dhaka, Bangladesh; icddr,b, Dhaka, Dhaka, Bangladesh; Charles Sturt University, New South Wales, New South Wales, Australia; icddr,b International Centre for Diarrhoeal Disease Research, Bangladesh, Dhaka, Dhaka, Bangladesh; icddr,b, Dhaka, Dhaka, Bangladesh; icddr,b, Dhaka, Dhaka, Bangladesh; icddr,b International Centre for Diarrhoeal Disease Research, Bangladesh, Dhaka, Dhaka, Bangladesh; icddr,b, Dhaka, Dhaka, Bangladesh; icddr,b International Centre for Diarrhoeal Disease Research, Bangladesh, Dhaka, Dhaka, Bangladesh; icddr,b, Dhaka, Dhaka, Bangladesh; icddr,b International Centre for Diarrhoeal Disease Research, Bangladesh, Dhaka, Dhaka, Bangladesh; icddr,b, Dhaka, Dhaka, Bangladesh; icddr,b, Dhaka, Dhaka, Bangladesh; icddr,b International Centre for Diarrhoeal Disease Research, Bangladesh, Dhaka, Dhaka, Bangladesh; icddr,b International Centre for Diarrhoeal Disease Research, Bangladesh, Dhaka, Dhaka, Bangladesh; Institute of Epidemiology, Disease Control & Research IEDCR, Dhaka, Dhaka, Bangladesh; Institute of Epidemiology, Disease Control and Research IEDCR, Dhaka, Dhaka, Bangladesh; Centers for Disease Control and Prevention CDC, Atlanta, Georgia; US CDC, Atlanta, Georgia; icddr,b International Centre for Diarrhoeal Disease Research, Bangladesh, Dhaka, Dhaka, Bangladesh; icddr,b International Centre for Diarrhoeal Disease Research, Bangladesh, Dhaka, Dhaka, Bangladesh; Institute of Epidemiology, Disease Control and Research IEDCR, Dhaka, Dhaka, Bangladesh; Centers for Disease Control and Prevention CDC, Atlanta, Georgia; icddr,b International Centre for Diarrhoeal Disease Research, Bangladesh, Dhaka, Dhaka, Bangladesh

## Abstract

**Background:**

The hospital-based Nipah virus (NiV) sentinel surveillance is Bangladesh approximately misses half of NiV outbreaks in communities. Such limitation potentially delays outbreak response and control measures. This community-based study explored the healthcare-seeking pattern of individuals suffering from Nipah-like symptoms and the factors influencing them.

**Figure 1:**
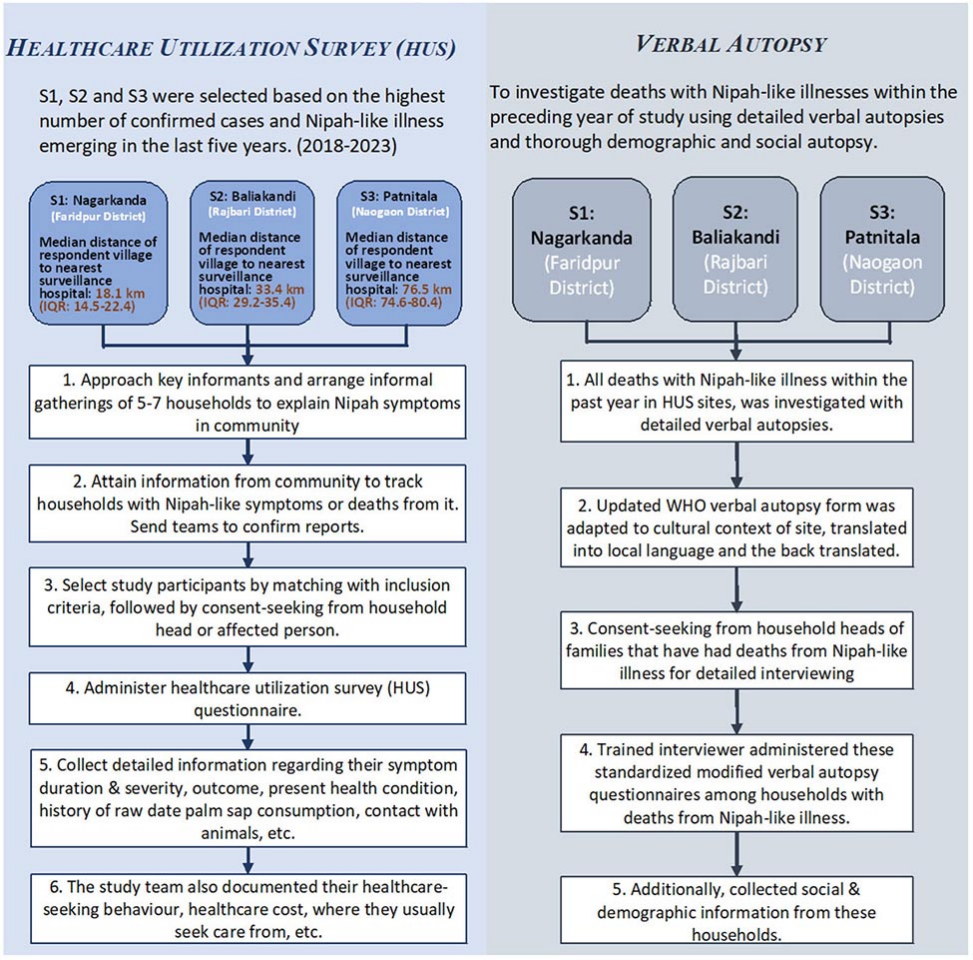
Overview of study methodology: site selection, healthcare utilization survey procedures, and verbal autopsy process

**Methods:**

From May to September 2023, we conducted a cross-sectional survey covering 176,648 households across three NiV-endemic sub-districts. Trained field team initially approached community key persons, including local health care providers, religious and community leaders, teachers and local vendors. Then they moved throughout the villages, arranged courtyard meetings with family key persons to identify individuals with active or past symptoms consistent with NiV infection, using a standardised case definition. Verbal autopsies (VA) were conducted for suspected NiV-related deaths (Fig. 1). Multivariable logistic regression was used to predict determinants of care-seeking.

**Results:**

Among 125 eligible individuals with Nipah-like illness, approximately 68% did not visit a Nipah-surveillance hospital, and 24% sought care from informal or unqualified healthcare providers. The individuals from sub-district S2 were 8.2 times more likely to visit a surveillance hospital compared to those from S1 (adjusted odds ratio [aOR]: 8.21, 95% CI: 2.16-31.13). Those with a monthly family income of ≤ $82 were about 20 times more likely to seek care at the surveillance hospital than those earning ≥$82 (aOR: 0.05, 95% CI: 0.00-0.54). Additionally, healthcare costs ($164-410) during the illness were associated with a sevenfold greater odds of visiting a surveillance hospital compared to costs of ≤ $164, suggesting individuals with more severe disease were more likely to seek care at these tertiary hospitals. Eight Niv-suspected deaths were identified, including one veterinary doctor. VA confirmed that three of them had symptoms compatible with acute meningo-encephalitis.

**Conclusion:**

Our findings suggest many individuals with Nipah-like illnesses remain undetected as they might not seek care, highlighting the need for a community-centred approach to enhance early detection and strengthen outbreak preparedness.

**Disclosures:**

All Authors: No reported disclosures

